# Neural repair in the adult brain

**DOI:** 10.12688/f1000research.7459.1

**Published:** 2016-02-12

**Authors:** Sebastian Jessberger

**Affiliations:** 1 Laboratory of Neural Plasticity, Brain Research Institute, Faculty of Medicine and Science, University of Zurich, Zurich, Switzerland

**Keywords:** Neural repair, brain injury, stem cell, Neural stem cell, neurogenesis

## Abstract

Acute or chronic injury to the adult brain often results in substantial loss of neural tissue and subsequent permanent functional impairment. Over the last two decades, a number of approaches have been developed to harness the regenerative potential of neural stem cells and the existing fate plasticity of neural cells in the nervous system to prevent tissue loss or to enhance structural and functional regeneration upon injury. Here, we review recent advances of stem cell-associated neural repair in the adult brain, discuss current challenges and limitations, and suggest potential directions to foster the translation of experimental stem cell therapies into the clinic.

## Introduction

Similar to the rest of the body, the brain is constantly at risk of damage through either acute or chronic injury. The long-standing assumption has been that the capacity for regeneration is strongly limited in the adult mammalian brain compared with other tissues such as the skin, liver, or intestines. In line with this, the mammalian brain is not able to simply regrow lost structures that are damaged during deleterious events such as ischemic stroke or traumatic brain injury. However, there is substantial functional restoration with acute or chronic injury because of the ability of surviving neural structures to take over at least partially the previous functions of lost tissues. This becomes clear, for example, with patients who have left-hemispheric strokes and may initially suffer from motor or sensory aphasia: with extensive training and rehabilitation, a substantial number of patients regain their ability to speak and communicate ^1^. Similarly, the brain can compensate functionally for massive loss of neural tissue before the consequences become apparent ^2^. For example, it is considered that more than 80% of all dopaminergic neurons in the substantia nigra are lost before Parkinsonian symptoms appear ^3^. Thus, the restorative potential of the adult mammalian brain to repair itself—at least functionally—certainly exists.

However, at the same time, these endogenous repair mechanisms have clear limitations, leaving a substantial percentage of patients with acute or chronic injury of the adult brain with permanent functional deficits. Thus, novel strategies need to be developed to ameliorate the course of degenerative or traumatic brain diseases. Substantial efforts have been made to either recruit or enhance endogenous repair mechanisms or to ameliorate brain function in the disease context by providing exogenous cells using transplantation ^4, 5^. Here, we focus exclusively on current approaches and ideas for how endogenous neural stem cells (NSCs) or other neural cells may be used to enhance brain repair.

## Neurogenic permissiveness in the adult brain

Already in the mid-1960s, first reports suggested that the generation of neurons in the mammalian brain is not limited to embryonic or early postnatal periods but that the adult brain retains the capacity to generate new neurons ^6– 8^. These findings were met with large skepticism because they challenged a long-standing dogma in the neurosciences stating that no new neurons may be born after the end of embryonic and early postnatal development ^9, 10^. It took another 30 years and the advent of novel techniques to unequivocally identify newborn neurons in the adult brain before the process of lifelong neurogenesis in the mammalian brain became broadly accepted ^11, 12^. However, the generation of new neurons is not widespread but appears to be restricted to distinct areas of the adult brain. One of those regions is the hippocampus, a key brain structure that, simplified, serves to regulate the sorting of certain experiences into long- and short-term memory and that has been identified as a neurogenic area permitting for the lifelong addition of dentate gyrus (DG) granule cells ^13, 14^.

The finding that NSCs persist even in the adult brain spurred tremendous efforts with the aim to recruit endogenous NSCs for enhanced brain repair upon injury. In addition, the fact that new neurons that are born throughout life with the possibility to functionally integrate into pre-existing circuitries gave rise to new hope that restoration of neural circuits via transplantation approaches using exogenous NSCs or other neural cells may be feasible in principle. Here, we review multiple facets of how stem cell-associated processes may be harnessed for future regenerative approaches. Furthermore, we discuss how characterizing the neurogenic process in the adult brain may help to improve our understanding of disease etiology and progression and how this understanding may help to develop novel strategies to treat diseases of the adult brain.

## Targeting endogenous neurogenesis for brain repair

NSCs generate new neurons in discrete regions of the adult brain ^15^. In the rodent brain, two main neurogenic areas have been identified. One of those is the subventricular zone (SVZ) lining the lateral ventricles where NSCs give rise to newborn cells that migrate along the rostral migratory stream toward the olfactory bulb (OB), where they differentiate into different types of olfactory neurons ^16, 17^. Whereas SVZ/OB neurogenesis is very substantial in the rodent brain, the neurogenic activity of the SVZ seems extremely reduced or absent in the human brain ^18, 19^. This is in contrast to the second main neurogenic area: the hippocampal DG, where NSCs give rise throughout life to DG granule cells ^20^. Multiple lines of evidence suggest that also in the human hippocampus a substantial number of neurons are born throughout life and that as a result a substantial part of the DG granule cell population is generated during postnatal life ^21, 22^.

At the top of the neurogenic lineage stand largely quiescent NSCs, called type-B cells in the SVZ and type-1 or radial glia-like NSCs in the DG, that have a number of astrocytic properties (such as expression of astroglial markers and vascular end-feet) ^16, 23, 24^. Upon activation, through extrinsic and intrinsic signals, radial glia-like NSCs enter the cell cycle and give rise to more proliferative type-C cells (SVZ) or type-2 cells (DG) generating neuroblasts that eventually differentiate into newborn neurons and integrate into the DG or OB circuits over the course of several weeks ^25– 30^. Notably, the levels of neurogenesis in the adult brain are dynamically regulated with a number of positive (e.g., physical activity, learning, and environmental enrichment) and negative (e.g., stress, aging, and inflammation) regulators through a number of intrinsic and extrinsic factors ^13, 31– 40^. Furthermore, it has been shown that hippocampal neurogenesis is substantially altered in a number of animal disease models ^13^. For example, neurogenesis is reduced in animal models of major depression but enhanced upon treatment with certain antidepressants such as selective serotonin reuptake inhibitors (SSRIs) ^41– 44^. Strikingly, adult hippocampal neurogenesis seems to be required for at least some aspects of the antidepressant efficacy of SSRIs in animal models of depression ^45^. These effects may not strictly qualify as “neural repair”, but it is reasonable to consider endogenous neurogenesis, in this case enhanced through antidepressants, as support of the improperly functioning brain to rebuild its correct connectivity.

Another example where altered neurogenesis in the adult DG may contribute to the disease process is temporal lobe epilepsy (TLE). In animal models of TLE, substantial changes in the levels of neurogenesis (acutely: enhanced; chronically: reduced) and strongly abnormal modes of neuronal integration (i.e. aberrant migration and ectopic synapse formation) have been described, suggesting that seizure-induced neurogenesis may contribute to the process of epileptogenesis and TLE-associated co-morbidities such as cognitive impairment ^46– 52^. However, there is also evidence that altered neurogenesis in animal models of TLE may rather represent an attempt of the injured brain to repair itself by balancing excess excitation that occurs in animal models of TLE ^53^. Be that as it may, targeting neurogenesis either to prevent abnormal and ectopic neurogenesis or to foster regenerative neurogenesis in the context of TLE may reduce the development of seizures or reduce co-morbidities associated with later stages of TLE such as hippocampus-dependent cognitive decline ^54^.

Furthermore, a large number of chronic neurodegenerative diseases, such as Alzheimer’s disease, have been associated with reduced neurogenesis that may participate in the progression of observed behavioral phenotypes of these diseases ^13^. Thus, future approaches will aim to enhance neurogenesis with the goal of either partially stopping disease progression or ameliorating secondary cognitive symptoms.

Without any doubt, the basic understanding of the function of adult neurogenesis needs to be characterized in much more detail to understand its potential role in disease processes. Newborn neurons are involved in a number of learning tasks and cognitive processes ^55– 59^. In addition, there is now ample evidence that hippocampal neurogenesis is also associated with emotional control ^60, 61^. However, it is still rather unclear when and how new neurons fulfill their action, even though accumulating data suggest that the period of heightened excitability may be critical for the effects of adult-born neurons on circuit activity ^27, 28, 62– 64^. Notably, it is currently believed not just that the purpose of hippocampal neurogenesis is simply to replace other neurons but that the key function of neurogenesis may be to provide young and excitable new neurons ^65^. This may be in contrast to lifelong neurogenesis in the SVZ/OB, where, in rodents, the continuous generation of new neurons is also critically involved in proper tissue homeostasis ^66^. Despite recent progress ^67, 68^, we still miss mechanistic data explaining the role of physiological neurogenesis that may be important to guide future experiments with the aim to harness the endogenous stem cell-associated potential for neural repair.

Overall, the role for endogenous SVZ/OB neurogenesis in the context of human neural repair is less clear given the strong evidence that the neurogenic niche in the human SVZ underlies very substantial changes during the early postnatal periods that may not support lifelong neurogenesis ^19, 69^. Furthermore, novel technological approaches (such as C14-based birth dating of neurons) suggest that no or only extremely few new neurons are integrated into the human OB during adulthood ^18^. In addition, ischemic stroke (that is sufficient to trigger at least transient neurogenesis in the rodent striatum) apparently does not lead to substantial formation of new neurons in the human cortex ^70– 72^. However, these findings clearly do not rule out the potential for SVZ-associated neurogenesis also in the human brain. In fact, a recent study showed that, despite the virtual absence of neurogenesis in the human OB, substantial numbers of newborn neurons could be detected in the human striatum that become substantially depleted in patients with Huntington’s disease, suggesting that neurogenesis outside the DG and SVZ/OB may be involved in human disease ^73^. However, it appears that newborn striatal neurons are generated by local neurogenic astroglial cells and are not derived by NSCs residing in the SVZ ^73– 75^. Future studies will have to aim to identify the molecular and cellular details of striatal neurogenesis in rodents and humans.

## Neural stem cell-based glial repair

Apart from approaches aiming to enhance endogenous neurogenesis for neuronal repair in the context of acute or chronic disease, the fate potential of endogenous NSCs also permits for targeting NSCs to support glial cell replacement and subsequent neural repair. In the rodent SVZ, it has been shown, for example, that demyelination leads to enhanced NSC-derived generation of oligodendrocytes that may help to remyelinate the injured brain upon lesion ^76, 77^. Similarly, induced generation of oligodendrocytes (that are not generated by DG NSCs under normal conditions) may represent an approach to induce remyelination of the DG circuitry for several demyelinating diseases such as multiple sclerosis or epilepsy ^78– 81^. However, potential therapeutic strategies aiming to use endogenous NSCs for glial repair are currently only beginning to be developed and additional evidence for their efficacy to improve brain function needs to be generated in rodent models of human disease.

## Inducing neurogenesis outside the neurogenic niches

Physiological neurogenesis from NSCs may be extremely restricted in the human brain—potentially exclusively to the DG under physiological conditions. However, recent evidence has shown that neural cells that are non-neurogenic under normal conditions may be amenable to exogenous reprogramming cues, allowing them to generate neuronal cells *in vivo*. This hypothesis was initially based on the fact that NSCs share many molecular and cellular features with classical astrocytes that are found throughout the brain parenchyma ^16, 82, 83^. Indeed, there is now compelling evidence that providing appropriate transcriptional cues is sufficient to induce neurogenesis throughout the cortex and other regions of the central nervous system ^84– 93^.

At this time, the main target population to ectopically induce the generation of newborn neurons are astrocytes. However, there is also evidence that other glial cells such as oligodendrocytes, oligodendrocyte precursor cells, or pericytes may be targeted to induce neurogenesis throughout the brain ^93– 98^. Apart from testing different cellular populations in the injured brain that may be used to generate new neurons, the generation of neuronal subtypes is a key interest in the field with the idea to replace the exact neuronal subtype that may be preferentially lost in certain diseases (e.g., dopaminergic neurons in the context of Parkinson’s disease) ^97, 99^. These experiments are guided by pioneering work studying the mechanisms controlling brain development using cocktails or sequential overexpression of subtype-specific transcription factors. Furthermore, current studies attempt to translate data that were generated by using *in vitro* fate specification approaches into the *in vivo* situation ^97, 100^. Thus, inducing neurogenesis with high spatial control in injured brain areas may represent a promising approach for targeted brain repair.

## Key challenges and future directions

**Figure 1.**
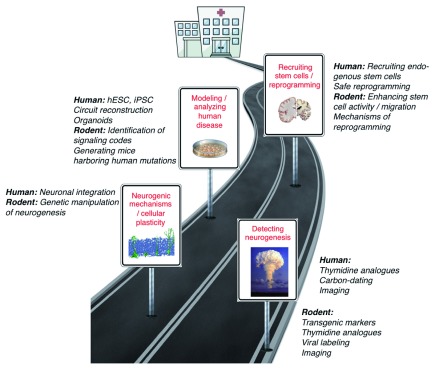
Road to harnessing stem cells and reprogramming strategies for neural repair. Future experiments will relate basic research findings obtained mostly in laboratory animals to the analyses of human disease and eventually to the therapeutic targeting of endogenous neural stem cells, the improved use of transplantation-based cell replacement strategies, or the reprogramming of other neural cells with the aim to enhance the potential for repair of the adult human brain. The road toward translation may lead from understanding physiologic and disease-associated neurogenesis in humans and an improved understanding of the molecular and cellular mechanisms underlying the neurogenic process toward novel approaches to study human diseases in the dish and mouse models. Finally, the application of this knowledge may lead to enhanced recruitment of endogenous stem cells or improved functionality of transplants and reprogramming-based approaches for neural repair. hESC, human embryonic stem cell; iPSC, induced pluripotent stem cell.

The finding that NSCs persist in the brain throughout life has been the starting point for novel approaches to enhance brain repair ( Figure 1). The applications for targeting NSCs are manifold and range from their potential involvement in the disease process (e.g., major depression) to their ability to generate new neuronal and glial cells (e.g., neurodegenerative diseases such as Alzheimer’s disease). In addition, approaches to induce neurogenesis outside physiological neurogenic niches may be of translational value ^5, 101^. However, we are still only beginning to understand what it takes for new neurons to truly make a functional impact on the injured brain. Key to improving these approaches will be to identify the mechanisms that regulate meaningful and proper integration into existing circuitries. This may be more feasible for some diseases where neurons may rather fulfill the function of providing neurotransmitters such as dopamine, but potentially more challenging and further away from clinical applications if diffuse circuitries or complete brain areas are impaired or destroyed.

Apart from increasing our understanding of the potential of endogenous NSCs or other neural cells for brain repair, the detailed molecular and cellular characterization of these processes also may be helpful to guide and improve current attempts to ameliorate brain function upon injury using exogenous transplantation of NSCs or other neural cells ^4, 101^. The key questions, such as neuronal differentiation, control of growth, and proper neuronal integration, are shared between these two strategies (NSC activation versus transplantation-based approaches) to target endogenous neural cells and to support brain repair with exogenous cells. In addition, it is foreseeable that ongoing studies aiming to understand disease processes using human embryonic stem cells or induced pluripotent stem cell-based approaches not only will improve our understanding of disease mechanisms but also may guide future strategies to enhance endogenous neural repair.

## Abbreviations

DG, dentate gyrus; NSC, neural stem cell; OB, olfactory bulb; SSRI, selective serotonin reuptake inhibitor; SVZ, subventricular zone; TLE, temporal lobe epilepsy.
